# Non-Random Distribution of G-Quadruplex Structures Reveals Regulatory and Ecological Adaptations in Bacterial Genomes

**DOI:** 10.3390/ijms262010025

**Published:** 2025-10-15

**Authors:** Jiye Fu, Ke Xiao, Yukun He, Jing Tu

**Affiliations:** 1 State Key Laboratory of Digital Medical Engineering, School of Biological Science and Medical Engineering, Southeast University, Nanjing 210096, Chinaykhe@seu.edu.cn (Y.H.); 2 Institute of Microphysiological Systems, School of Biological Science and Medical Engineering, Southeast University, Nanjing 210096, China

**Keywords:** G-quadruplexes, bacterial genome, ecological analysis, transcriptional regulation

## Abstract

G-quadruplexes (G4s) are guanine-rich DNA secondary structures with known regulatory roles in eukaryotes, but their functions in bacteria remain largely unexplored. Here, we systematically analyzed potential G-quadruplexes (PG4s) across 1566 bacterial genomes. PG4s showed non-random distributions, with strong enrichment in intergenic regions, particularly near gene start sites, suggesting potential roles in transcriptional regulation. Structural analysis revealed a higher proportion of short-loop G4s in these regions, indicating enhanced stability. Comparisons with randomized genomes confirmed that the observed patterns cannot be explained by GC content or genome size alone. Ecological analysis further revealed that bacteria associated with homothermic hosts exhibit significantly lower G4 densities than free-living species, suggesting reduced reliance on G4-mediated regulation in stable environments. Together, these findings highlight G4s as conserved yet context-dependent elements of bacterial genome organization and provide new insights into their potential regulatory roles in prokaryotes.

## 1. Introduction

G-quadruplexes (G4s) are non-canonical nucleic acid secondary structures formed by guanine-rich sequences that fold into four-stranded conformations stabilized by Hoogsteen hydrogen bonding and the presence of monovalent cations such as potassium or sodium [1]. These unique structures have attracted increasing scientific attention due to their distinctive topological properties and their potential to influence a variety of biological processes [2,3]. Over the past two decades, studies in eukaryotic systems have revealed that G4s are far from being rare structural curiosities: they are abundant across genomes and are enriched at functionally significant loci such as promoters, telomeres, untranslated regions, and replication origins [4,5,6]. Experimental evidence has firmly established that G4s in eukaryotes can regulate transcription, replication, genome stability, and chromatin organization [7,8]. Their functional versatility, together with their frequent localization in key regulatory regions, has spurred intense interest in exploring them as potential therapeutic targets, particularly in cancer biology, virology, and neurodegenerative diseases [9,10,11].

By comparison, our understanding of G4 biology in bacteria remains fragmentary. Early computational surveys identified guanine-rich motifs with the potential to form G4s across bacterial genomes, suggesting that these structures are not confined to eukaryotes [12]. Subsequent biochemical assays have confirmed that bacterial sequences are indeed capable of forming stable G4 structures in vitro [13,14]. Furthermore, a handful of studies have linked specific G4 motifs to important bacterial traits, such as phase variation in surface antigens, virulence regulation, and stress responses [15,16]. For example, in *Neisseria gonorrhoeae*, G4 elements have been implicated in antigenic variation that facilitates immune evasion [17]. In *Mycobacterium tuberculosis*, G4 structures have been suggested to modulate the expression of genes involved in pathogenicity [18]. Despite these intriguing findings, the scope of bacterial G4 research remains narrow. Most existing studies have focused on a limited set of species, particularly well-studied model organisms and clinically relevant pathogens [14,19,20,21]. This bias leaves the vast diversity of bacteria, including environmental isolates, commensals and symbionts, largely unexplored with respect to their potential G4 repertoires. Consequently, our current knowledge reflects only a small fraction of bacterial diversity and may not capture the broader evolutionary or ecological roles of G4s. Moreover, while isolated examples suggest functional significance, the general principles that govern the occurrence, genomic distribution, and biological relevance of G4s in bacterial genomes have yet to be systematically defined. Without such a framework, it remains unclear whether bacterial G4s are incidental structural motifs or conserved elements shaped by selective pressures.

One reason for this knowledge gap is that bacterial regulatory systems differ fundamentally from those of eukaryotes. Bacteria lack chromatin and nucleosomes, and their genomes are highly compact, with limited intergenic regions and frequent operonic organization. These features constrain the regulatory space available for DNA secondary structures [22,23]. At the same time, bacteria are extraordinarily diverse and adaptable organisms, inhabiting ecological niches that range from hydrothermal vents and saline lakes to plant roots and animal guts. Their rapid growth rates, high mutation frequencies, and capacity for horizontal gene transfer drive evolution on timescales far shorter than those observed in eukaryotes [24,25]. As a consequence, bacterial genomes are exquisitely shaped by environmental pressures, and regulatory innovations that confer adaptive advantages can become fixed rapidly. If G4s participate in gene regulation in bacteria, their distribution is likely to be influenced by ecological and evolutionary constraints.

Temperature represents one of the most fundamental environmental parameters affecting bacterial survival. Genome composition, codon usage, and protein stability are all known to vary with optimal growth temperature [26,27,28]. It is therefore reasonable to hypothesize that G4 motifs, if functionally relevant, might also exhibit temperature-associated patterns. Similarly, host association provides another axis of selective pressure. Bacteria living in the relatively constant environment of homothermic animal hosts experience markedly different challenges from those encountered by free-living bacteria exposed to fluctuating environmental conditions [29,30]. Yet, to date, no large-scale study has systematically evaluated how such ecological and evolutionary pressures shape the distribution of G4 motifs in bacterial genomes [14,31]. Addressing this gap is crucial for determining whether G4s in bacteria are incidental products of sequence composition or represent adaptive features with functional roles.

Here, we present a systematic investigation of G4 distribution across a broad spectrum of bacterial genomes from a population-level perspective. By integrating large-scale bioinformatic predictions with ecological metadata, we reveal non-random patterns of G4 occurrence and provide evidence that their prevalence and genomic positioning are shaped by both intrinsic sequence properties and ecological contexts.

## 2. Results

### 2.1. Overall Characteristics of the Bacterial Dataset

After stringent filtering and selection, we assembled a dataset consisting of 1566 representative bacterial genomes from 41 different phyla, along with their corresponding gene annotations. The distribution of species across phyla is summarized in Supplementary Table S1. Genome sizes in this dataset ranged from 510 kbp (*Mycoplasmopsis felis*) to 13.66 Mbp (*Streptomyces species*), reflecting the wide diversity of bacterial genome architectures. The GC content of these genomes spanned from 24.44% (*Mycoplasmopsis felis*) to 76.16% (*Miltoncostaea marina*), capturing both AT-rich and GC-rich extremes of bacterial genomic diversity (Supplementary Table S2).

**Figure 1:**
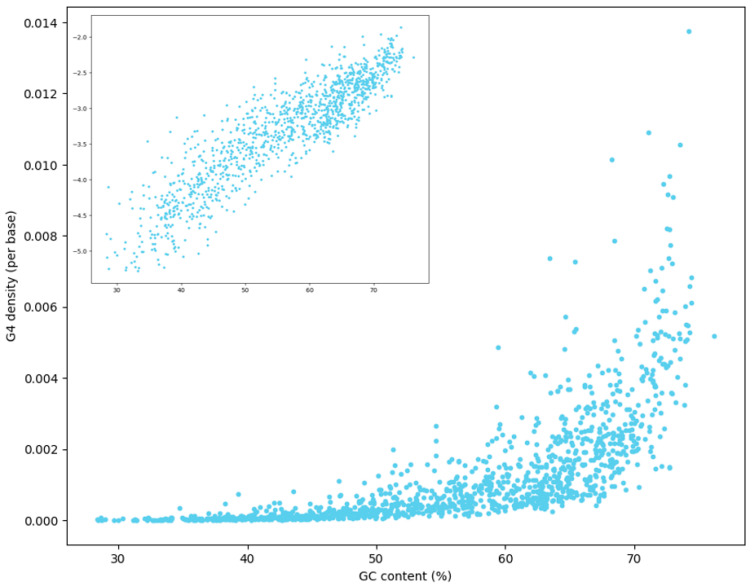
Density of G4 structures across bacterial genomes with varying GC content. Each point represents a bacterial species. The main plot shows G4 density (number of G4s per base of reference genome length) plotted against genomic GC content. The embedded subplot in the upper left displays the same data with a logarithmic *y*-axis to better visualize variation in genomes with low G4 density and the exponential relationship between GC content and G4 density.

Using a computational prediction pipeline based on canonical G4 motifs, we identified a total of 404,608 PG4s across all genomes. The relationship between PG4 density and genomic GC content is shown in Figure 1. A clear positive correlation was observed, with PG4 density increasing exponentially with higher GC content. This trend is consistent with the intrinsic sequence composition of G4 motifs, which require multiple guanine tracts, and therefore are more likely to occur in GC-rich genomes. Importantly, the exponential association between GC content and PG4 density agrees with findings from earlier large-scale bacterial genome analyses, reinforcing the notion that GC content is the primary determinant of G4 abundance in bacterial genomes. However, the large taxonomic breadth of our dataset allowed us to confirm this relationship at an unprecedented scale, across more than 40 bacterial phyla. It should be noted that noncanonical G4s, including bulged or two-tetrad motifs, have been experimentally observed in bacterial genomes. While these were not analyzed here, future studies integrating such variants could further refine our understanding of G4 diversity and function in prokaryotes.

### 2.2. Enrichment of G4s in Specific Genomic Regions of Bacteria

**Figure 2:**
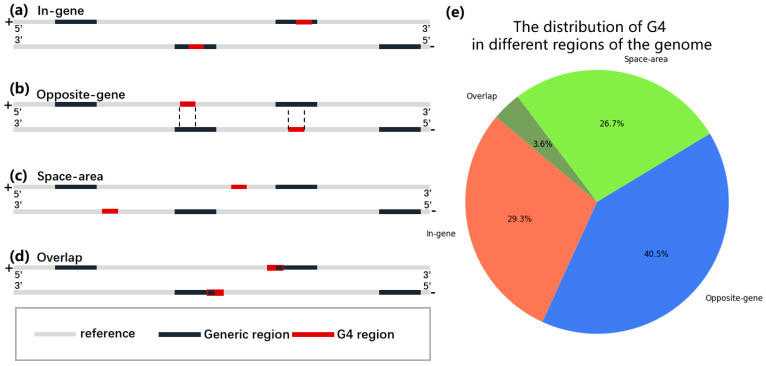
Four classifications of G4 defined based on different overlapping relationships between G4 and genomic annotations and their quantitative statistics. (**a**) In-gene G4s, entirely located within gene region. (**b**) Opposite-gene G4s, entirely located on the strand opposite to a gene region. (**c**) Space-area G4s, located within intergenic regions on both strands. (**d**) Overlapping G4s, spanning both gene and intergenic regions or their antisense counterparts. (**e**) The proportion of G4s from these four different regions in all G4 of the genome.

The formation of G4 structures within bacterial genomes has the potential to influence not only the expression of the genes in which they occur but also that of the corresponding complementary strand. To account for this, both strands of each genome were included in the annotation process when classifying G4s. As shown in Figure 2, G4s were classified by the method described in Section 4.3, and notable differences in G4 distribution were observed between the coding strand and its complementary counterpart, despite the fact that these two regions share identical total lengths. Specifically, approximately 40% of PG4s were classified as opposite-gene G4s, whereas only ~26% were located within gene-coding regions (in-gene G4s). This marked asymmetry suggests that G4s tend to occur more frequently in non-coding contexts relative to protein-coding sequences, possibly reflecting selective pressure against the presence of stable secondary structures within actively transcribed regions. In addition, a substantial fraction of PG4s (30.2%) were found in intergenic (space-area) regions, despite these regions accounting for only 13.8% of the total genome length. This overrepresentation is striking and strongly indicates that intergenic regions serve as preferred sites for G4 formation. In conventional bacterial genome annotation, the regions defined here as “opposite-gene” also overlap with intergenic segments, further supporting the idea that the regulatory potential of G4s in bacteria is largely concentrated in non-coding regions of the genome. Such enrichment may facilitate transcriptional regulation by providing structural elements in promoter- or terminator-proximal regions, where dynamic DNA conformations could influence gene expression and genome organization.

**Figure 3:**
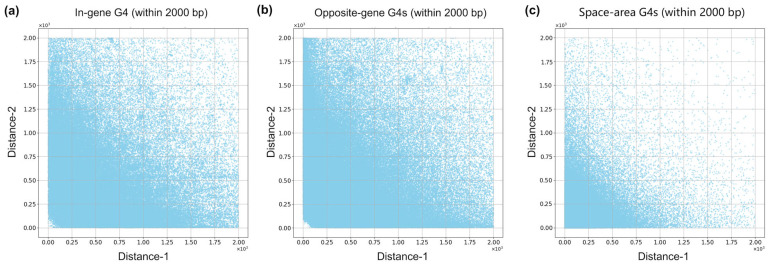
Spatial distribution of G4s relative to region boundaries in three genomic contexts. Three scatter plots depict the positions of individual G4s within (**a**) in-gene, (**b**) opposite-gene, and (**c**) space-area regions. For each G4, the horizontal axis (Distance 1) and vertical axis (Distance 2) represent its distance to the two boundaries of the region in which it is located. Only data points where both Distance 1 and Distance 2 are within 2000 bp are shown. “Distance 1” and “Distance 2” represent the distances from each G4 motif to the two boundaries of the genomic region in which it is located. Smaller values indicate G4s positioned closer to region boundaries, reflecting their potential clustering near gene starting or termini.

To further explore this hypothesis, we examined the spatial distribution of G4s within different genomic regions (Figure 3). Strikingly, most PG4s located in intergenic (space-area) regions tended to cluster near the boundaries of adjacent coding sequences. By contrast, PG4s within in-gene or opposite-gene regions displayed a more uniform distribution across their respective regions. This observation suggests that G4s in intergenic areas are not randomly positioned, but instead exhibit a targeted enrichment near gene boundaries, consistent with potential roles in transcriptional initiation or regulatory control. Quantitative evaluation using the *C*-value defined in Section 4.5 further supported this pattern: values of 0.45 for in-gene G4s, 0.21 for opposite-gene G4s, and 0.72 for space-area G4s confirmed the strong positional bias of intergenic G4s.

**Figure 4:**
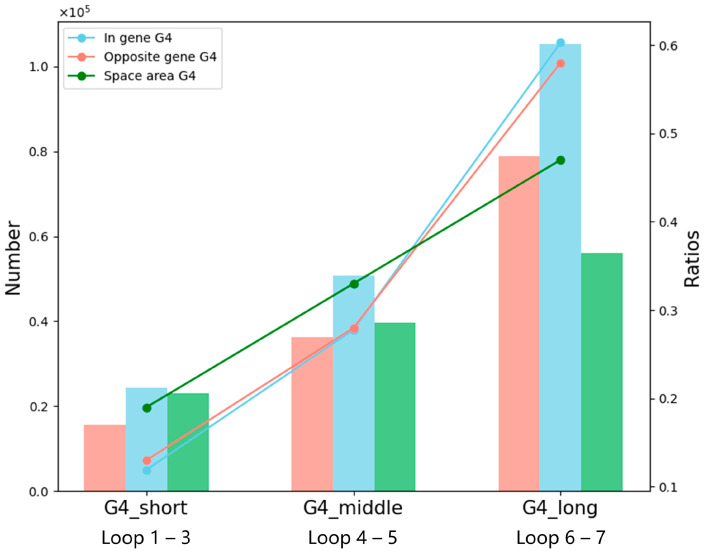
Distribution and relative abundance of G4s by loop length categories across different genomic regions. This hybrid bar and line plot displays the loop length distribution of G4s located in in-gene, opposite-gene, and space-area regions. The left *y*-axis represents the absolute counts of G4s (bar height), while the right *y*-axis indicates the proportional ratio among the three loop types within each region. Bars and lines are colored according to genomic region (in-gene, opposite-gene, space-area), facilitating comparison of both absolute quantities and normalized distribution ratios across regions.

Loop length is another important determinant of G4 stability, and therefore we analyzed the distribution of loop-length categories in different genomic contexts, as shown in Figure 4. For G4s located in in-gene and opposite-gene regions, the proportions of short-loop, medium-loop, and long-loop G4s followed an approximately 2:4:5 ratio, indicating a balanced distribution. In contrast, G4s within intergenic (space-area) regions showed a markedly different profile (3:3:4). The major difference lays in the increased proportion of short-loop G4s, which are known to form more stable quadruplex structures and occur with lower probability under random nucleotide arrangements. The relative enrichment of short-loop G4s in intergenic regions therefore underscores their potential biological significance, suggesting that space-area G4s are selectively maintained and may serve as robust structural elements in gene regulation.

To ensure that these findings were not artifacts of bacterial genome size or sampling bias, we compared the observed distributions with those from randomly generated genomes. Artificial genomes of 2 Mb length were simulated across GC contents ranging from 45% to 70% (no substantial G4s were detectable at GC contents below 40%, and thus these cases were omitted), as shown in Supplementary Figure S1. In all cases, replicate simulations produced nearly identical numbers of G4s, and the loop-length distributions remained stable at approximately 2:4:5, closely matching the ratios observed in in-gene and opposite-gene regions of real bacterial genomes (Supplementary Figure S2). This concordance suggests that G4s within protein-coding regions largely conform to expectations of random distribution.

**Figure 5:**
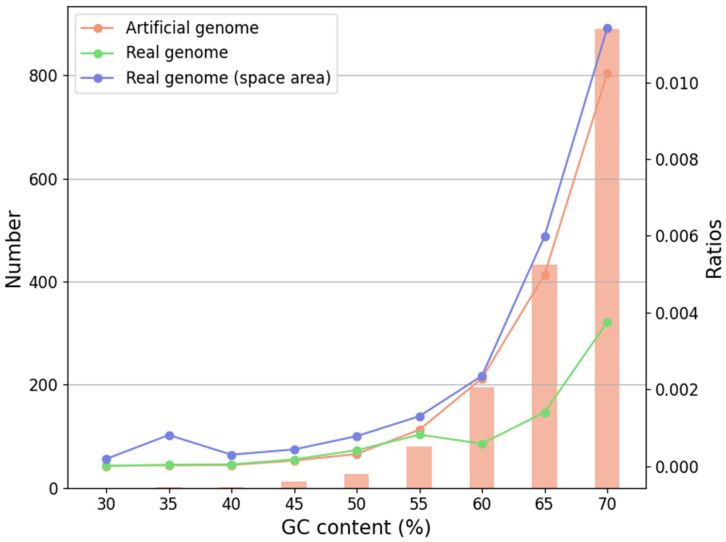
Comparison of G4 abundance and density between synthetic and real bacterial genomes across varying GC content. GC content is binned in intervals of 5%, ranging from 30% to 70%. The orange bars (left *y*-axis) represent the absolute number of G4s detected in artificially generated genomes. Line plots illustrate G4 density (number of G4s per unit length, right *y*-axis) for three datasets: artificially generated genomes (orange), real bacterial genomes (cyan), and the space-area (intergenic) regions within real bacterial genomes.

However, further comparison of G4 densities between artificial and real genomes revealed an important distinction, as shown in Figure 5. At GC contents below 55%, the density of G4s in real bacterial genomes was only slightly higher than in randomized controls. In contrast, above 55% GC, bacterial genomes consistently exhibited lower G4 densities than their artificial counterparts, suggesting selective suppression of G4 formation in high-GC coding contexts. Notably, intergenic G4s deviated from this trend: across all GC contents, space-area G4s were significantly more abundant in real bacterial genomes than in random controls, reinforcing the conclusion that intergenic enrichment is a genuine and biologically meaningful feature rather than a compositional artifact. Collectively, these analyses demonstrate that the enrichment of G4s near gene boundaries and the elevated proportion of stable short-loop G4s in intergenic regions represent robust and reproducible patterns. These features distinguish functional G4 distribution in bacterial genomes from random expectations, highlighting the selective importance of G4s in intergenic contexts as potential regulatory elements in bacterial gene expression.

### 2.3. Host Environment as a Key Determinant of Bacterial G4 Distribution

**Figure 6:**
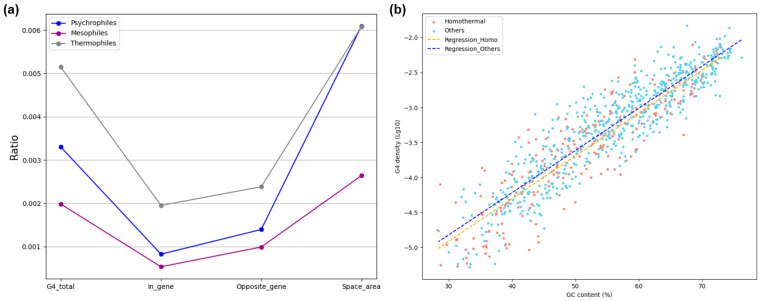
Environmental and genomic correlates of G4 density across bacterial groups. (**a**) G4 density in different genomic regions for psychrophilic, mesophilic, and thermophilic bacteria. Line plots compare the G4 density among psychrophilic, mesophilic, and thermophilic bacterial groups, illustrating variation in G4 abundance relative to optimal growth temperature. (**b**) Relationship between GC content and G4 density in bacteria from homothermic hosts and the others. Linear regression lines are fitted to each category to highlight trends in G4 density as a function of GC content.

To further investigate the diversity of G4 distribution among bacteria, we categorized bacterial genomes according to their optimal growth temperature (based on BacDive annotations), roughly classifying them into psychrophiles, mesophiles, and thermophiles. We first analyzed the relationship between G4 occurrence and different genomic regions, as shown in Figure 6a. Consistent with previous results, G4s were most frequently located in intergenic regions, followed by opposite-gene regions, and least frequently within genes. Given that G4 is a temperature-sensitive DNA secondary structure, we expected that the overall density of G4s in bacterial genomes would vary in a linear manner with increasing growth temperature. However, the statistical analysis did not support this hypothesis. Instead of a monotonic trend, mesophilic bacteria showed the lowest G4 density among the three groups. We hypothesized that this unexpected pattern was not coincidental but rather related to whether bacteria inhabit homothermic animal hosts. To test this, we divided bacterial genomes into two groups: (i) host-associated bacteria that parasitize homothermic animals, and (ii) free-living bacteria broadly distributed in natural environments. The log-scaled distribution of G4 densities for the two groups is shown in Figure 6b. Variance homogeneity analysis confirmed no significant difference in variance between the two groups (*p* = 0.681 > 0.05), allowing a valid t-test. The result revealed a significant difference in mean G4 density (the *p*-value of the *t*-test is 0.005).

Linear regression analysis further supported this conclusion, with high coefficients of determination (R^2^ = 0.84 for host-associated bacteria and R^2^ = 0.74 for free-living bacteria). The two regression lines were nearly parallel, indicating that across a wide range of genomic GC content, host-associated bacteria consistently exhibited lower G4 density compared to free-living bacteria. The intercept difference (~0.1 in log scale) translates to an approximately 20% reduction in G4 density in host-associated bacteria at the same GC content. This population-wide reduction in G4 density suggests that bacterial genomes associated with homothermic hosts harbor fewer regulatory elements, likely due to the relatively stable thermal environment provided by their hosts. Consequently, these bacteria may rely more on host physiology for adaptation and less on intrinsic genomic regulatory mechanisms. Moreover, bacteria inhabiting homothermic hosts exhibit a lower proportion of short-loop G4 structures, suggesting a more limited functional diversity of G4 motifs in these genomes (Supplementary Figure S3).

In summary, while the distribution of G4s across genomic regions remains consistent among bacteria, their overall density is strongly shaped by ecological context rather than temperature alone. The reduced G4 abundance in host-associated bacteria highlights the influence of stable host environments on genomic regulatory complexity, underscoring the adaptive role of G4s in free-living species that face more variable conditions.

While our results highlight a clear reduction in G4 density among host-associated bacteria, this observation may partly reflect phylogenetic composition and genome reduction. Host-associated species often belong to specific bacterial lineages and exhibit smaller, AT-rich genomes, both of which can intrinsically lower G4 abundance. However, the consistent difference we observed across varying GC contents, and even among closely related genera within the same family (where one genus is host-associated and the other is free-living), suggests that this trend is not merely a taxonomic artifact. Nevertheless, we recognize that ecological interpretations should be approached with caution, and our analysis provides one perspective among several possible explanations for the observed distribution patterns.

## 3. Discussion

Our systematic investigation of potential G-quadruplexes (PG4s) across bacterial genomes provides new insights into their non-random distribution and functional implications. We demonstrated that G4 motifs are preferentially enriched in intergenic and opposite-gene regions, particularly in space-area regions adjacent to coding sequences. This positional bias, together with the higher prevalence of short-loop G4s, suggests that these structures may contribute to fine-tuned regulation of transcription initiation rather than being stochastic by-products of genome composition. Comparative analyses with randomized genomes further confirmed that these enrichments are not artifacts of sampling or GC content but reflect selective constraints in bacterial genomes.

In addition, the regulatory potential of G4s may also depend on their structural topology, such as parallel or antiparallel strand orientations and distinct tetrad arrangements, which were not considered in the present sequence-based analysis [32,33]. Future studies integrating structural characterization could provide deeper insight into how these topological variations contribute to functional diversity among bacterial G4s.

Moreover, our cross-species analysis revealed ecological patterns shaping G4 distribution. Specifically, bacteria parasitic to homothermic hosts exhibit consistently lower G4 densities than free-living species, independent of genome GC content. This reduction likely reflects reduced reliance on G4-mediated regulatory mechanisms in environments with stable temperature and host-dependent metabolic support. In contrast, free-living bacteria, exposed to more variable conditions, appear to retain higher levels of G4 structural diversity and potential regulatory capacity.

Together, these findings highlight the evolutionary adaptability of G4 motifs in bacterial genomes. They not only enrich our understanding of DNA secondary structures as regulatory elements but also underscore their potential as novel targets for antibacterial strategies. It should be noted that only a limited number of bacterial G4 motifs have been experimentally confirmed to date, and the majority of predicted sequences remain unvalidated. Future work combining biochemical validation and functional assays will be critical to uncovering the mechanistic roles of bacterial G4s in gene regulation and environmental adaptation.

## 4. Materials and Methods

### 4.1. Bacterial Reference Genomes and Annotations

All bacterial reference genomes and their corresponding annotation files were obtained from the National Center for Biotechnology Information (NCBI) database, and taxonomic classification was assigned according to the current NCBI taxonomy system. To ensure representativeness, a unified selection criterion was applied at the genus level. Specifically, for each genus, the type species was first considered: if the genome assembly of the type species was available at the “complete genome” level, it was selected as the representative for that genus. If this requirement was not met, the species with the highest assembly quality within the genus was chosen instead. For genera in which all available genomes were below the chromosome level, the species with the highest assembly quality was retained only if the corresponding assembly was supported by more than 10 sequencing entries. Genera for which all genomes were below the scaffold level were excluded from the dataset. The designation of type species for each genus was referenced from the List of Prokaryotic names with Standing in Nomenclature (LPSN). In addition to the genus representatives selected by these rules, several widely studied bacterial species were also included to supplement the dataset.

### 4.2. Determination of Bacterial Habitats and Classification

The living conditions and isolation sources of each bacterium were determined primarily using information from the Bacterial Diversity database (BacDive) and the Japan Collection of Microorganisms (JCM) database. When these resources did not provide sufficient details to classify growth temperature or isolation source, relevant information was retrieved from published scientific literature. If no reliable data on growth environment or isolation source could be obtained through any of these channels, the corresponding bacterium was excluded from subsequent environmental and ecological classifications.

### 4.3. Prediction and Classification of Bacterial Genomic G4s

Putative G-quadruplexes (pG4s) in bacterial genomes were predicted using a regular expression–based approach. The canonical motif was defined as “GxNyGxNyGxNyGx,” where x ≥ 3 and y ranges from 1 to 7. PG4s identified by this method were regarded as canonical G4s, and overlapping predictions were filtered to avoid redundancy. This criterion was chosen because canonical G4s are well characterized in both experimental and computational contexts and exhibit stable folding behavior in vitro and in vivo. Although other noncanonical forms such as bugled, long-loop, or two-tetrad G4s have been reported in bacteria, these were not included in the present large-scale analysis due to their structural variability and the lack of standardized prediction algorithms.

Based on loop length, pG4s were further classified into three categories: short-loop (maximum loop length 1–3 nt), middle-loop (4–5 nt), and long-loop (6–7 nt). To account for variation in genome assembly and annotation quality across species, the positional relationship of putative G4s (PG4s) to annotated genomic regions was simplified into four catdaegories, as illustrated in Figure 2a–d. PG4s that extended across more than two distinct genomic regions were excluded from analysis, as such cases were too rare for meaningful statistical evaluation.

### 4.4. Generation of Random Genomes

Random genome sequences were generated using a Python 3.7 script implementing a shuffling algorithm, with each sequence set to a length of 2 Mb. The GC content of the sequences ranged from 30% to 70% in increments of 5%. For each GC content level, ten replicates were produced to minimize statistical fluctuations introduced by random sequence generation.

The artificial genomes were generated to serve as randomized controls, enabling us to test whether the observed patterns of G4 distribution in real bacterial genomes can be explained simply by nucleotide composition (e.g., GC content) and genome length, or whether they reflect non-random, biologically constrained organization.

### 4.5. Quantitative Evaluation of the Position Between G4s and Coding Regions

The distribution of G4s within genomic regions was quantitatively assessed using the following algorithm:

First, the length of each genomic region was normalized to 1. For each G4 located in a given region, the relative starting position was denoted as $x_{i}$ (0 ≤ $x_{i}$ ≤ 1), where 0 and 1 represent the boundaries of the region. The distances from $x_{i}$ to the two boundaries can be expressed as $d_{0}\left(x_{i}\right)\;={\;x}_{i}$ and $d_{1}\left(x_{i}\right)\;=\;1-x_{i}$. The minimum of these two values was taken as the effective distance to the nearest boundary:

$$d_{i}\;=\;\min(x_{i},\;1\;-\;x_{i})$$

If G4s were uniformly distributed within the region, the expected mean distance to the nearest boundary would be:

$$E\left[d\right]=\int_{0}^{0.5}xdx+\int_{0.5}^{1}\left(1-x\right)dx=\frac{1}{8}+\frac{1}{8}=\frac{1}{4}$$

The observed mean distance was calculated as

$$\bar{d}=\frac{1}{n}\sum_{i=1}^{n}d_{i}=\frac{1}{n}\sum_{i=1}^{n}min(x_{i},1-x_{i})$$

To quantify the relative positional bias, the observed mean distance was compared with the expected distance and normalized to the range [−1, 1] by defining the coefficient C:

$$C=1-\frac{\bar{d}}{E\left[d\right]}=1-\frac{\bar{d}}{0.25}=1-4\cdot \bar{d}$$

A higher C value (close to 1) indicates that G4s tend to cluster near the boundaries of the region, while a lower C value (close to −1) suggests enrichment near the center. When G4s are randomly distributed, $\bar{d}$ approaches $E\left[d\right]$, and C approaches 0. Statistical analyses in this study were primarily descriptive and correlation-based; no multiple testing corrections were applied.

## Data Availability

The original contributions presented in this study are included in the article/Supplementary Materials. Further inquiries can be directed to the corresponding author.

## Supplementary Materials

The following supporting information can be downloaded at https://www.mdpi.com/article/10.3390/ijms262010025/s1.
