# Supplementary material for: Non-Random Distribution of G-Quadruplex Structures Reveals Regulatory and Ecological Adaptations in Bacterial Genomes
Source: Int J Mol Sci. 2025 Oct 15;26(20):10025. doi: 10.3390/ijms262010025 (PMC12563890; doi:10.3390/ijms262010025)
Supplement: Supplementary file 1 [file ijms-26-10025-s001.zip › ijms-3912171-supplementary.pdf]

Supplementary Data

Non-Random Distribution of G-Quadruplex Structures Reveals Regulatory and Ecological Adaptations in Bacterial Genomes

Jiye Fu [1,†], Ke Xiao [1,†], Yukun He [1] and Jing Tu [1,2,*]

1 State Key Laboratory of Digital Medical Engineering, School of Biological Science and Medical Engineering, Southeast University, Nanjing 210096, China
2 Institute of Microphysiological Systems, School of Biological Science and Medical Engineering, Southeast University, Nanjing 210096, China
* Correspondence: jtu@seu.edu.cn
† The authors contribute equally to this work.

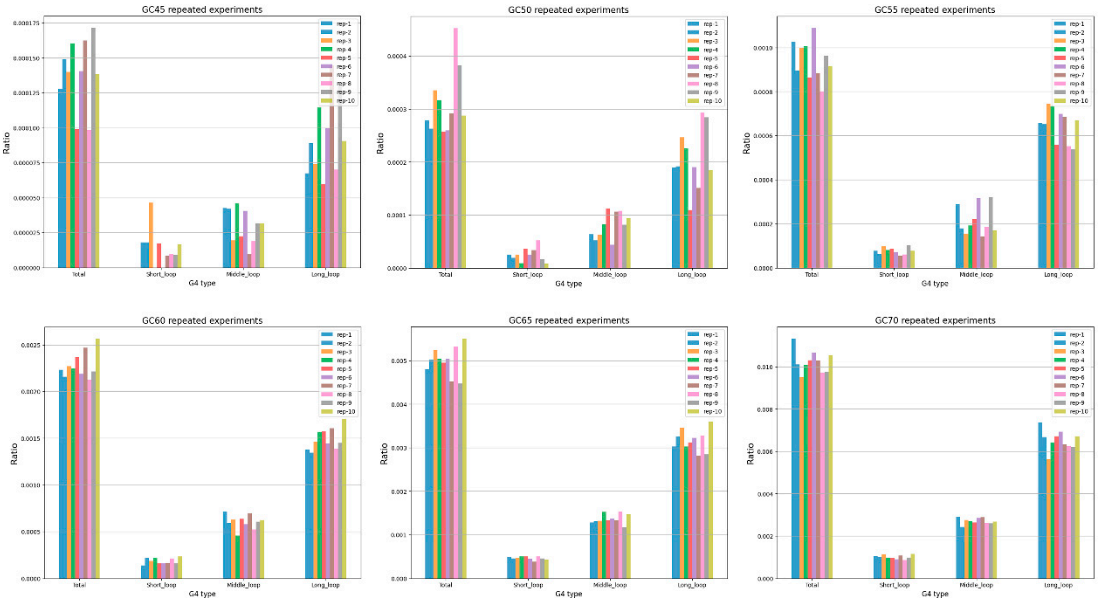

Figure S1. G4 density in artificially generated genomes across different GC contents. Six panels (GC content from 45% to 70%, in 5% increments) show the distribution of G4 density in synthetic genomes. For each GC bin, ten replicate genomes were generated. In each subplot, the x-axis represents four genomic categories: G4_total, In_gene, Opposite_gene, and Space_area. The y-axis indicates G4 density.

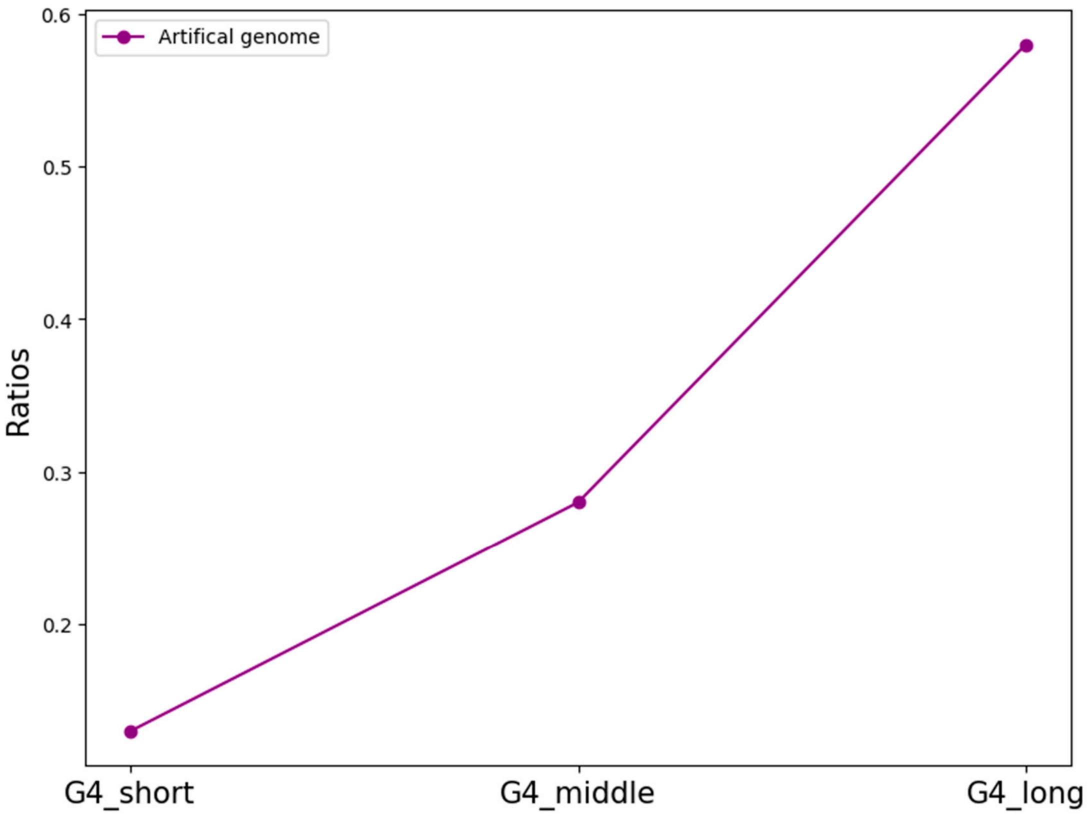

Figure S2. Proportion of G4 types by loop length in artificially generated genomes. The line plot displays the density of G4 structures classified into three loop-length categories: G4_short, G4_middle, and G4_long. The x-axis represents these G4 types, and the y-axis shows the corresponding G4 density. The trend illustrates the relative abundance of each G4 type across synthetic genomes.

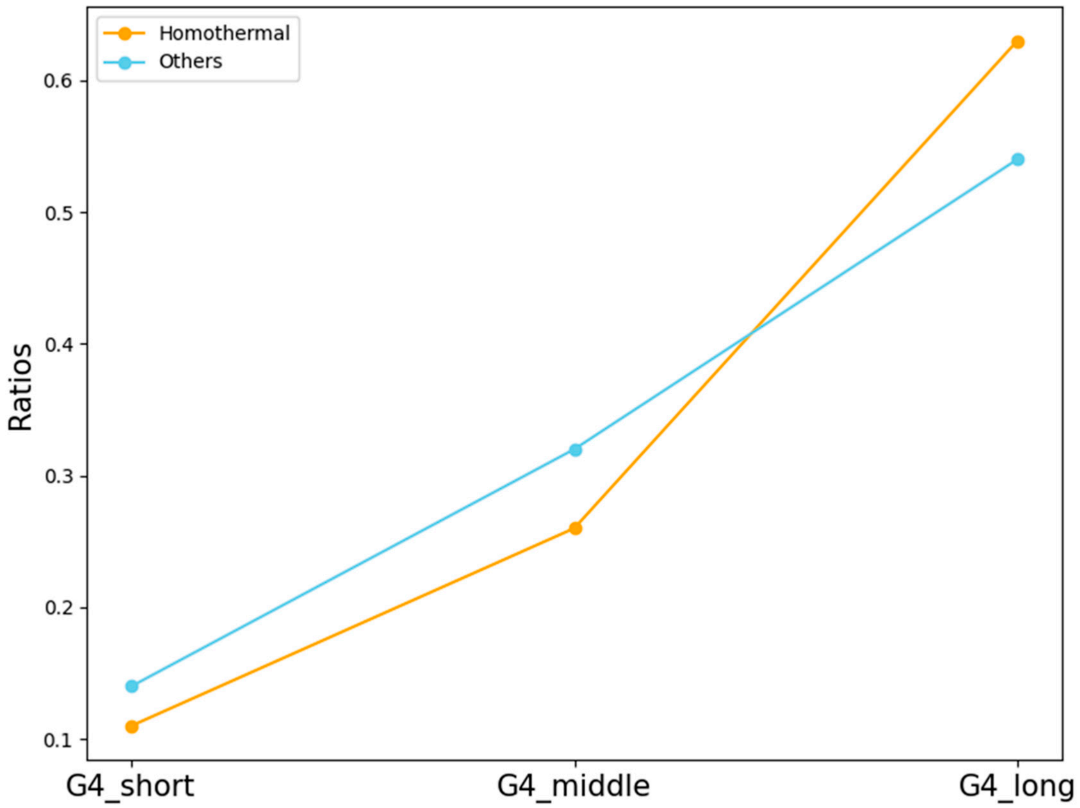

Figure S3. Comparative distribution of G4 loop types in bacteria from homeothermic versus other bacteria. This line plot illustrates the density of G4 structures categorized by loop length (G4_short, G4_middle, G4_long) across bacterial groups. The x-axis denotes the three G4 types, while the y-axis represents G4 density. The orange line corresponds to bacteria associated with homeothermic hosts, and the light blue line represents bacteria from other hosts.

Key process of the data analysis

1. Prediction of G4s:

run_g4predict.sh

```
for element in `folder of all bacteria`
do
    g4predict intra -f /`folder of all bacteria`/${element}/*.fna -b /`folder of all bacteria`/${element}/genome_PQ.bed -s -F
done
```

2. Classification of PG4s:

classified_by_GC.py

```
import os
def get_dirname(directory):
    dir_names = []
    names = os.listdir(directory)
    for name in names:
        if len(name.split('.')) == 1:
            dir_names.append(directory+name+'/')
        else:
            pass
    return dir_names
def get_filename(directory,suffix):
    names = os.listdir(directory)
    for name in names:
        if name.endswith(suffix):
            return name
def get_gc(ref_file):
    rfile = open(ref_file,'rt')
    lines = rfile.readlines()
    dic = {}
    total_base = 0
    GC_base = 0
    for line in lines:
        if line.startswith('>'):
            ref_name = line.strip().split(' ')[0].split('>')[-1]
            dic[ref_name] = ''
        else:
            dic[ref_name] += line.strip()
            total_base += len(line.strip())
```

```
                GC_base += line.count('G')+line.count('C')

        GC_content = GC_base/total_base *100
        return GC_content

if __name__ == '__main__':
        outfile1 = open('PQ_0-40.bed','wt')
        outfile2 = open('PQ_40-50.bed','wt')
        outfile3 = open('PQ_50-60.bed','wt')
        outfile4 = open('PQ_60-70.bed','wt')
        outfile5 = open('PQ_70-100.bed','wt')
        directory = ''
        dir_names = get_dirname(directory)
        for dir_name in dir_names:
                pq_file = dir_name + get_filename(dir_name,'genome_PQ.bed')
                ref_file = dir_name + get_filename(dir_name,'.fna')
                infile = open(pq_file,'rt')
                lines = infile.readlines()
                GC_content = get_gc(ref_file)
                if GC_content < 40:
                        for line in lines:
                                outfile1.writelines(line)
                elif GC_content >= 40 and GC_content < 50:
                        for line in lines:
                                outfile2.writelines(line)
                elif GC_content >= 50 and GC_content < 60:
                        for line in lines:
                                outfile3.writelines(line)
                elif GC_content >= 60 and GC_content < 70:
                        for line in lines:
```

```
            outfile4.writelines(line)
elif GC_content >= 70:
        for line in lines:
            outfile5.writelines(line)
else:
            print('Something wrong!')
```

3. Statistics of PQ locations

PQ_location.py

```
import os
def location_stat(lists,pq,total_length):
    '''
    Determine the relationship between a given G4 and the target regions:
    1. The input lists is an even-numbered list, where every two numbers form a pair — the odd-indexed element represents the start position, and the even-indexed element represents the end position.
    2. pq is a list where pq[0] is the start position and pq[1] is the end position.
    3. total_length refers to the chromosome length.
    '''
    temp_s = pq[0]
    temp_e = pq[1]
    location_list = []

    # First, check whether the list is empty.
    if lists == []:
        status = 0
        to_lower = 0
        to_higher = 0
    # Determine the state of pq when it is located at the beginning or end of the list
    elif temp_s <= lists[0] and temp_e > lists[0]:
        status = 2
        to_lower = temp_s
        to_higher = lists[1] - temp_e
    elif temp_e <= lists[0]:
        status = 0
        to_lower = temp_s
        to_higher = lists[0] - temp_e
```

```
    elif temp_e >= lists[-1] and temp_s < lists[-1]:
        status = 3
        to_lower = temp_s - lists[-2]
        to_higher = total_length - temp_e
    elif temp_s >= lists[-1]:
        status = 0
        to_lower = temp_s - lists[-1]
        to_higher = total_length - temp_e
    else:
        # Determine the start position of pq and calculate the distance between the start of pq and the end of the previous region.
        for i in range(len(lists)):
            if lists[i] <= temp_s:
                pass
            else:
                lower_num = lists[i-1]
                list_pos1 = i
                to_lower = temp_s - lower_num
                break
        # Determine the end position of pq and calculate the distance between the end of pq and the start of the next region.
        for j in range(len(lists)):
            if lists[j] < temp_e:
                pass
            else:
                higher_num = lists[j]
                list_pos2 = j+1
                to_higher = higher_num - temp_e
                break
        if list_pos2 - list_pos1 == 1 and list_pos1%2 == 0:
```

```
            status = 0
        elif list_pos2 - list_pos1 == 1 and list_pos1%2 == 1:
            status = 1
        elif list_pos2 - list_pos1 == 2 and list_pos1%2 == 0:
            status = 2
        elif list_pos2 - list_pos1 == 2 and list_pos1%2 == 1:
            status = 3
        elif list_pos2 - list_pos1 > 2:
            status = 4
        else:
            print('Something wrong!')
    location_list = [status,to_lower,to_higher]
    return location_list

def gff_dic_form(infile):
    dic = {}
    i = 0
    list_plus = []
    list_minus = []
    if infile.endswith('.gz') or infile.endswith('.gzip'):
        inf = gzip.open(infile,'rt')
    else:
        inf = open(infile,'rt')
    lines = inf.readlines()
    for line in lines:
        if not line.startswith('#'):
            li = line.strip().split('\t')
            if li[2] == 'region':
                if i == 0:
                    i = 1
```

```
                else:
                        dic[ref_name] = [total_length,list_plus,list_minus]
                        list_plus = []
                        list_minus = []
                total_length = int(li[4])
                ref_name = li[0]
        elif li[2] == 'gene':
                if li[6] == '+':
                        list_plus.append(int(li[3]))
                        list_plus.append(int(li[4]))
                else:
                        list_minus.append(int(li[3]))
                        list_minus.append(int(li[4]))
    dic[ref_name] = [total_length,list_plus,list_minus]
    return dic

def PQ_split(string):
    loops = string.split('b')[-1].split('l')[0].split(',')
    if int(loops[0]) <= 3 and int(loops[1]) <= 3 and int(loops[2]) <= 3:
        split_result = 'S'
    elif int(loops[0]) <= 5 and int(loops[1]) <= 5 and int(loops[2]) <= 5:
        split_result = 'M'
    else:
        split_result = 'L'
    return split_result

def PQ_measure(pqfile,dic,ref_file,outfile):
    outf = open(outfile,'wt')
    dic_ref = get_refseq(ref_file)
    infile = open(pqfile,'rt')
```

```
lines = infile.readlines()
print('Now at:' + ref_file.split('/')[-2])
if len(lines) == 0:
    print('No g4 in :'+pqfile)
else:
    for line in lines:
        lin = line.strip()
        li = lin.split('\t')
        ref_name = li[0]
        start = int(li[1])
        end = int(li[2])
        string = li[3]
        chain = li[5]

        split_result = PQ_split(string)
        total_length = dic[ref_name][0]
        lists_plus = dic[ref_name][1]
        lists_minus = dic[ref_name][2]
        pq = [start,end]
        pq_string = dic_ref[ref_name][start:end]
        location_list_plus = location_stat(lists_plus,pq,total_length)
        location_list_minus = location_stat(lists_minus,pq,total_length)
        if chain == '+' and location_list_plus[0] == 1:
            stat = 'in_gene'
        elif chain == '-' and location_list_minus[0] == 1:
            stat = 'in_gene'
        elif chain == '+' and location_list_minus[0] == 1:
            stat = 'opposite_gene'
        elif chain == '-' and location_list_plus[0] == 1:
            stat = 'opposite_gene'
```

```
                    elif location_list_plus[0] == 0 and location_list_minus[0] == 0:
                        stat = 'space_area'
                    elif location_list_plus[0] == 1 and location_list_minus[0] == 1:
                        stat = 'cross_area'
                    else:
                        stat = 'overlap'
                    str_list                                                    =
[ref_name,li[1],li[2],string,pq_string,chain,split_result,str(location_list_plus[0]),str(lo
cation_list_plus[1]),str(location_list_plus[2]),str(location_list_minus[0]),str(location_l
ist_minus[1]),str(location_list_minus[2]),stat]
                    new_line = '\t'.join(str_list)
                    new_line += '\n'
                    outf.writelines(new_line)

def get_refseq(ref_file):
    rfile = open(ref_file,'rt')
    lines = rfile.readlines()
    dic = {}
    for line in lines:
        if line.startswith('>'):
            ref_name = line.strip().split(' ')[0].split('>')[-1]
            dic[ref_name] = ''
        else:
            dic[ref_name] += line.strip()
    return dic

def get_dirname(directory):
    dir_names = []
    names = os.listdir(directory)
    for name in names:
```

```
            if len(name.split('.')) == 1:
                if name == 'README' or name == 'README~':
                    pass
                else:
                    dir_names.append(directory+name+'/')
            else:
                pass
    return dir_names

def get_filename(directory,suffix):
    names = os.listdir(directory)
    for name in names:
        if name.endswith(suffix):
            return name
if __name__ == '__main__':
    directory = 'folder of all bacteria'
    dir_names = get_dirname(directory)
    for dir_name in dir_names:
        gff_file = dir_name + get_filename(dir_name,'.gff')
        pq_file = dir_name + get_filename(dir_name,'PQ.bed')
        out_file = dir_name + 'PQ_with_tag.bed'
        ref_file = dir_name + get_filename(dir_name,'.fna')
        dic = gff_dic_form(gff_file)
        PQ_measure(pq_file,dic,ref_file,out_file)
```
